# Supplementary material for: SARS-CoV-2 infections in migrants and the role of household overcrowding: a causal mediation analysis of Virus Watch data
Source: J Epidemiol Community Health. 2023 Jul 18;77(10):649–55. doi: 10.1136/jech-2022-220251 (PMC10511992; doi:10.1136/jech-2022-220251)
Supplement: Supplementary data [file jech-2022-220251supp001.pdf]

Contents

Supplementary Box 1: DAGitty code 2

Supplementary Box 2: Occupation categories 3

Supplementary Box 3: Causal mediation analysis and multicollinearity checks 4

Supplementary Box 4: Variables used for multiple imputation 7

Supplementary Table 1: STROBE checklist (cohort study specific) 8

Supplementary Table 2: Cohort demographic and household characteristics 11

Supplementary Table 3: Percentage of participants with evidence of SARS-CoV-2 infection in the second wave (1 September 2020 to 30 April 2021) 17

Supplementary Figure 1: Directed acyclic graph of the relationship between migration status and SARS-CoV-2 infection 21

Supplementary Figure 2: Counts for different person per room (PPR) values across the study cohort 22

References 23

Boukari Y, *et al. J Epidemiol Community Health* 2023; 77:649–655. doi: 10.1136/jech-2022-220251

Supplementary Box 1: DAGitty code

```
dag {
bb="-5.865,-4.971,4.732,9.643"
"Age, sex and ethnicity" [adjusted,pos="-5.064,1.174"]
"Clinical vulnerability" [pos="-0.043,3.758"]
"Geographic region" [pos="-4.772,-3.687"]
"Living with children" [pos="0.279,0.247"]
"Migration status" [exposure,pos="-4.293,7.497"]
"SARS-CoV-2 infection" [outcome,pos="3.048,7.676"]
"Self selection" [latent,pos="-1.569,6.484"]
Income [pos="-2.460,-2.281"]
Occupation [pos="1.566,-3.947"]
Overcrowding [pos="3.707,-2.419"]
"Age, sex and ethnicity" -> "Clinical vulnerability"
"Age, sex and ethnicity" -> "Living with children"
"Age, sex and ethnicity" -> "Migration status"
"Age, sex and ethnicity" -> "SARS-CoV-2 infection"
"Age, sex and ethnicity" -> "Self selection"
"Age, sex and ethnicity" -> Income
"Age, sex and ethnicity" -> Occupation
"Age, sex and ethnicity" -> Overcrowding
"Clinical vulnerability" -> "SARS-CoV-2 infection"
"Clinical vulnerability" -> "Self selection"
"Geographic region" -> Income
"Geographic region" -> Occupation
"Geographic region" -> Overcrowding
"Living with children" -> "SARS-CoV-2 infection"
"Living with children" -> Occupation
"Migration status" -> "Clinical vulnerability"
"Migration status" -> "Geographic region"
"Migration status" -> "Living with children"
"Migration status" -> "SARS-CoV-2 infection"
"Migration status" -> "Self selection"
"Migration status" -> Income
"Migration status" -> Occupation
"Migration status" -> Overcrowding
Income -> "Clinical vulnerability"
Income -> "SARS-CoV-2 infection"
Income -> Overcrowding
Occupation -> "SARS-CoV-2 infection"
Occupation -> Income
Occupation -> Overcrowding
Overcrowding -> "SARS-CoV-2 infection"
}
```

Supplementary Box 2: Occupation categories

- Administrative and secretarial
- Healthcare
- Indoor trades, process and plant
- Leisure and personal service
- Managers, directors and senior officials
- Other professional and associate
- Outdoor trades
- Sales and customer service
- Social care and community protective services
- Teaching, education and childcare
- Transport and mobile machine
- Not in employment

Supplementary Box 3: Causal mediation analysis and multicollinearity checks

An observational study can generally be conceptualised as a conditionally randomised experiment if four key identifiability conditions are met [1]. We outline these conditions and how they relate to our study in the table below.

Condition	Met	Rationale
**No interference** (i.e. an individual's potential outcome does not depend on another individual's exposure)	Yes	Another individual being a migrant does not affect whether a UK-born individual gets infected.<br><br>However, a study in the US found higher rates of COVID-19 mortality in urban areas with more immigrants who had traditional/multigenerational family structures. Being a migrant is associated with an increased odds of infection and as SARS-CoV-2 is infectious, living in an area with more migrants could mean increased risk of infection for UK-born too.
**Consistency** (i.e. the effect of an exposure is the same for all individuals who receive that exposure)	Yes	Migration status is based on whether someone reports a UK or non-UK country of birth. It is not a composite variable.
**Positivity** (i.e. all individuals have a probability of greater than 0 of being assigned all values of the exposure variable in every stratum defined by covariates)	Yes	Theoretically possible for all.
**Conditional exchangeability** (i.e. all individuals with and without exposure are comparable in every respect apart from the exposure after	Yes	We controlled for age, sex, ethnicity, clinical vulnerability, household income, occupation and living with children status.

conditioning on the confounders)		

Traditional methods to decompose the total effect into direct and indirect effects involve comparing regression coefficients before and after controlling for the potential mediator; however, these methods are inappropriate for use with non-linear models [2]. The Buis method decomposes the total causal effect of a categorical variable within a logistic regression into indirect and direct effects, and it does not require a normally distributed mediator variable. It has also been applied to the Virus Watch cohort to decompose the total effect of occupation on SARS-CoV-2 serological status [3].

The direct effect was the association between migration status and SARS-CoV-2 infection due to all other causes not accounted for in the model. It was derived by comparing the proportion of migrants with evidence of infection with the counterfactual proportion of UK-born individuals who would have had evidence of infection if they had the same distribution of household overcrowding status as the migrant individuals i.e., household overcrowding status was kept constant. To estimate the direct effect, confounders of both the exposure-mediator, mediator-outcome and exposure-outcome mediator must be adjusted for. Based on the DAG in Supplementary Figure 1, the minimally sufficient adjustment set for the direct effect comprises age at baseline, sex at birth, ethnicity, clinical vulnerability, total household income at baseline and occupation.

The indirect effect was the association between migration status and SARS-CoV-2 infection due to differences in household overcrowding status. It is derived by comparing the proportion of migrants who had evidence of SARS-CoV-2 infection with the counterfactual proportion of migrants who would have had evidence of infection if they had the same distribution of household overcrowding status as UK-born participants, while adjusting for the same confounders that were adjusted for when estimating the direct effect. The total effect is estimated by summing the indirect and direct coefficients on the log scale.

Model checks for multicollinearity, particularly in the case of migration status and ethnicity, were conducted using logistic regression controlled for age, sex and ethnicity with the *glm* command in R version 4.1.2 with the family set to binomial and a logit link. The generalised variance inflation factor (GVIF) was calculated using the *vif* command in the *car* package [4] with adjustment for the number of coefficients in the variables [5]. GVIFs adjusted for the number of coefficients in the variables were all below 10 (Table below), which suggests a lack of evidence of multicollinearity [6].

Table: GVIFs adjusted for the number of coefficients in the variable for a regression model representing the total effect

Variable	GVIF adjusted for number of coefficients in the variable
UK born status	1.40
Age group	1.01
Sex	1.14
Ethnicity	1.12

Boukari Y, *et al. J Epidemiol Community Health* 2023; 77:649–655. doi: 10.1136/jech-2022-220251

Supplementary Box 4: Variables used for multiple imputation

The following variables were included as predictor variables:

- Index of multiple deprivation
- Household region
- Sex
- Age at study entry
- Ethnicity
- UK-born status
- Whether there are children living in the household
- Accommodation-related variables: type, self-contained status, presence of central heating, ownership/rental status, number of rooms in household and presence of damp or mould
- Household overcrowding status
- Combined household income
- Clinical vulnerability status
- Living with children
- Occupation
- Whether an individual had a positive test in the study period

Supplementary Table 1: STROBE checklist (cohort study specific)

	Item No	Recommendation	Page No
**Title and abstract**	1	(*a*) Indicate the study's design with a commonly used term in the title or the abstract	p1, 2
		(*b*) Provide in the abstract an informative and balanced summary of what was done and what was found	
**Introduction**			
Background/rationale	2	Explain the scientific background and rationale for the investigation being reported	p4
Objectives	3	State specific objectives, including any prespecified hypotheses	p4
**Methods**			
Study design	4	Present key elements of study design early in the paper	p5
Setting	5	Describe the setting, locations, and relevant dates, including periods of recruitment, exposure, follow-up, and data collection	p5
Participants	6	(*a*) Give the eligibility criteria, and the sources and methods of selection of participants. Describe methods of follow-up	p5
		(*b*) For matched studies, give matching criteria and number of exposed and unexposed	
Variables	7	Clearly define all outcomes, exposures, predictors, potential confounders, and effect modifiers. Give diagnostic criteria, if applicable	p5,6
Data sources/ measurement	8*	For each variable of interest, give sources of data and details of methods of assessment (measurement). Describe comparability of assessment methods if there is more than one group	p5,6
Bias	9	Describe any efforts to address potential sources of bias	p7
Study size	10	Explain how the study size was arrived at	p5
Quantitative variables	11	Explain how quantitative variables were handled in the analyses. If applicable, describe which groupings were chosen and why	p5,6
Statistical methods	12	(*a*) Describe all statistical methods, including those used to control for confounding	p6,7
		(*b*) Describe any methods used to examine subgroups and interactions	
		(*c*) Explain how missing data were addressed	
		(*d*) If applicable, explain how loss to follow-up was addressed	
		(*e*) Describe any sensitivity analyses	

Boukari Y, *et al. J Epidemiol Community Health* 2023; 77:649–655. doi: 10.1136/jech-2022-220251

**Results**			
Participants	13*	(a) Report numbers of individuals at each stage of study—eg numbers potentially eligible, examined for eligibility, confirmed eligible, included in the study, completing follow-up, and analysed	Figure 1, p8
		(b) Give reasons for non-participation at each stage	
		(c) Consider use of a flow diagram	
Descriptive data	14*	(a) Give characteristics of study participants (eg demographic, clinical, social) and information on exposures and potential confounders	Table 1, p10-16
		(b) Indicate number of participants with missing data for each variable of interest	
		(c) Summarise follow-up time (eg, average and total amount)	
Outcome data	15*	Report numbers of outcome events or summary measures over time	Table 2, p17<br>Table 3, p18-21

Main results	16	(*a*) Give unadjusted estimates and, if applicable, confounder-adjusted estimates and their precision (eg, 95% confidence interval). Make clear which confounders were adjusted for and why they were included	Table 4, p22
		(*b*) Report category boundaries when continuous variables were categorized	
		(*c*) If relevant, consider translating estimates of relative risk into absolute risk for a meaningful time period	
Other analyses	17	Report other analyses done—eg analyses of subgroups and interactions, and sensitivity analyses	p22,23
**Discussion**			
Key results	18	Summarise key results with reference to study objectives	p24
Limitations	19	Discuss limitations of the study, taking into account sources of potential bias or imprecision. Discuss both direction and magnitude of any potential bias	p24, 25
Interpretation	20	Give a cautious overall interpretation of results considering objectives, limitations, multiplicity of analyses, results from similar studies, and other relevant evidence	p24, 25
Generalisability	21	Discuss the generalisability (external validity) of the study results	p24, 25
**Other information**			
Funding	22	Give the source of funding and the role of the funders for the present study and, if applicable, for the original study on which the present article is based	p27

*Give information separately for exposed and unexposed groups.

Supplementary Table 2: Cohort demographic and household characteristics

Characteristic	Overall<br>N = 23,478	UK-born<br>N = 21,416	Migrant<br>N = 2,062
**Age**			
Mean (SD)	59 (15)	59 (15)	53 (16)
Median (IQR)	62 (19)	63 (19)	53 (26)
<30	1,379 (5.9%)	1,241 (5.8%)	138 (6.7%)
30-39	1,718 (7.3%)	1,341 (6.3%)	377 (18.3%)
40-49	2,537 (10.8%)	2,163 (10.1%)	374 (18.1%)
50-59	4,397 (18.7%)	4,022 (18.8%)	375 (18.2%)
60+	13,447 (57.3%)	12,649 (59.1%)	798 (38.7%)
**Sex**			
Female	12,810 (54.6%)	11,592 (54.1%)	1,218 (59.1%)
Male	10,276 (43.8%)	Omitted to avoid disclosure	Omitted to avoid disclosure
Other or unknown	392 (1.7%)	Omitted to avoid disclosure	Omitted to avoid disclosure
**Ethnicity**			

Characteristic	Overall<br>N = 23,478	UK-born<br>N = 21,416	Migrant<br>N = 2,062
White British	20,456 (87.1%)	19,972 (93.3%)	484 (23.5%)
Minority ethnic*	2,544 (10.8%)	986 (4.6%)	1,558 (75.6%)
Missing	478 (2.0%)	458 (2.1%)	20 (1.0%)
**Geographical region**			
East Midlands	2,057 (8.8%)	1,971 (9.2%)	86 (4.2%)
East of England	5,208 (22.2%)	4,889 (22.8%)	319 (15.5%)
London	2,933 (12.5%)	2,044 (9.5%)	889 (43.1%)
North East	1,153 (4.9%)	1,105 (5.2%)	48 (2.3%)
North West	2,500 (10.6%)	2,409 (11.2%)	91 (4.4%)
South East	4,524 (19.3%)	4,147 (19.4%)	377 (18.3%)
South West	1,752 (7.5%)	1,641 (7.7%)	111 (5.4%)
Wales	540 (2.3%)	521 (2.4%)	19 (0.9%)
West Midlands	1,276 (5.4%)	1,218 (5.7%)	58 (2.8%)
Yorkshire and The Humber	1,153 (4.9%)	1,106 (5.2%)	47 (2.3%)

Characteristic	Overall<br>N = 23,478	UK-born<br>N = 21,416	Migrant<br>N = 2,062
Missing	382 (1.6%)	365 (1.7%)	17 (0.8%)
**IMD quintile**			
1 (most deprived)	1,909 (8.1%)	1,679 (7.8%)	230 (11.2%)
2	3,384 (14.4%)	2,948 (13.8%)	436 (21.1%)
3	4,770 (20.3%)	4,297 (20.1%)	473 (22.9%)
4	6,124 (26.1%)	5,675 (26.5%)	449 (21.8%)
5 (least deprived)	6,909 (29.4%)	6,452 (30.1%)	457 (22.2%)
Missing	382 (1.6%)	365 (1.7%)	17 (0.8%)
**Total household income**			
£0-9,999	806 (3.4%)	712 (3.3%)	94 (4.6%)
£10,000-24,999	4,453 (19.0%)	4,158 (19.4%)	295 (14.3%)
£25,000-49,999	7,080 (30.2%)	6,570 (30.7%)	510 (24.7%)
£50,000-£74,999	3,568 (15.2%)	3,173 (14.8%)	395 (19.2%)
£75,000-£99,999	1,726 (7.4%)	1,504 (7.0%)	222 (10.8%)

Characteristic	Overall<br>N = 23,478	UK-born<br>N = 21,416	Migrant<br>N = 2,062
£100,000+	1,725 (7.3%)	1,410 (6.6%)	315 (15.3%)
Missing	4,120 (17.5%)	3,889 (18.2%)	231 (11.2%)
**Number of rooms in household**			
Mean (SD)	6 (2)	6 (2)	5 (2)
Median (IQR)	6 (2)	6 (2)	5 (3)
**Number of householders**			
1	4,640 (19.8%)	4,230 (19.8%)	410 (19.9%)
2	13,571 (57.8%)	12,538 (58.5%)	1,033 (50.1%)
3	2,647 (11.3%)	2,345 (10.9%)	302 (14.6%)
4	1,983 (8.4%)	1,738 (8.1%)	245 (11.9%)
5	540 (2.3%)	478 (2.2%)	62 (3.0%)
6	97 (0.4%)	87 (0.4%)	10 (0.5%)
**Household overcrowding status**			
Under occupied	21,134 (90.0%)	19,662 (91.8%)	1,472 (71.4%)

Characteristic	Overall<br>N = 23,478	UK-born<br>N = 21,416	Migrant<br>N = 2,062
Balanced	1,686 (7.2%)	1,320 (6.2%)	366 (17.7%)
Overcrowded	658 (2.8%)	434 (2.0%)	224 (10.9%)
**Persons per room**			
Mean (SD)	0.49 (0.33)	0.47 (0.29)	0.71 (0.53)
Median (IQR)	0.40 (0.31)	0.40 (0.29)	0.50 (0.67)
**Living with any children**			
No	20,246 (86.2%)	18,647 (87.1%)	1,599 (77.5%)
Yes	3,232 (13.8%)	2,769 (12.9%)	463 (22.5%)
**Occupation**			
Administrative and secretarial	1,378 (5.9%)	1,238 (5.8%)	140 (6.8%)
Healthcare	735 (3.1%)	629 (2.9%)	106 (5.1%)
Indoor trades, process and plant	660 (2.8%)	587 (2.7%)	73 (3.5%)
Leisure and personal Service	484 (2.1%)	412 (1.9%)	72 (3.5%)
Managers, directors and senior officials	855 (3.6%)	760 (3.5%)	95 (4.6%)

Characteristic	Overall<br>N = 23,478	UK-born<br>N = 21,416	Migrant<br>N = 2,062
Other professional and associate	3,345 (14.2%)	2,829 (13.2%)	516 (25.0%)
Outdoor trades	254 (1.1%)	232 (1.1%)	22 (1.1%)
Sales and customer service	521 (2.2%)	474 (2.2%)	47 (2.3%)
Social care and community protective services	543 (2.3%)	485 (2.3%)	58 (2.8%)
Teaching, education and childcare	1,150 (4.9%)	1,019 (4.8%)	131 (6.4%)
Transport and mobile machine	229 (1.0%)	210 (1.0%)	19 (0.9%)
Not in employment†	9,924 (42.3%)	9,247 (43.2%)	677 (32.8%)
Missing	3,400 (14.5%)	3,294 (15.4%)	106 (5.1%)
**Clinical vulnerability status**			
Not clinically vulnerable	12,964 (55.2%)	11,715 (54.7%)	1,249 (60.6%)
Clinically vulnerable	6,395 (27.2%)	5,846 (27.3%)	549 (26.6%)
Clinically extremely vulnerable	2,714 (11.6%)	2,470 (11.5%)	244 (11.8%)
Missing	1,405 (6.0%)	1,385 (6.5%)	20 (1.0%)

*All minority ethnic groups were combined to avoid statistical disclosure. †Represents individuals who are retired, students, looking after the household/family and not looking for work, permanently sick or disabled, or unemployed.

Supplementary Table 3: Percentage of participants with evidence of SARS-CoV-2 infection in the second wave (1 September 2020 to 30 April 2021)

	UK-born			Migrant		
		Evidence of SARS-CoV-2 infection			Evidence of SARS-CoV-2 infection	
**Characteristic**	**Overall, N=21,416[1]**	**No n=20,074 (93.7%)**	**Yes n=1,342 (6.27%)**	**Overall, N = 2,062**	**No n=1,875 (90.9%)**	**Yes n=187 (9.07%)**
**Age**						
<30	1,241	1,125 (90.7%)	116 (9.3%)	138	123 (89.1%)	15 (10.9%)
30-39	1,341	1,235 (92.1%)	106 (7.9%)	377	332 (88.1%)	45 (11.9%)
40-49	2,163	1,969 (91.0%)	194 (9.0%)	374	322 (86.1%)	52 (13.9%)
50-59	4,022	3,691 (91.8%)	331 (8.2%)	375	343 (91.5%)	32 (8.5%)
60+	12,649	12,054 (95.3%)	595 (4.7%)	798	755 (94.6%)	43 (5.4%)
**Sex**						
Male	9,435	8,877 (94.1%)	558 (5.9%)	841	770 (91.6%)	71 (8.4%)
Female	11,592	10,823 (93.4%)	769 (6.6%)	1,218	1,102 (90.5%)	116 (9.5%)
**Ethnicity**						
White British	19,972	18,723 (93.7%)	1,249 (6.3%)	484	451 (93.2%)	33 (6.8%)

17

	UK-born			Migrant		
		Evidence of SARS-CoV-2 infection			Evidence of SARS-CoV-2 infection	
**Characteristic**	**Overall, N=21,416[1]**	**No n=20,074 (93.7%)**	**Yes n=1,342 (6.27%)**	**Overall, N = 2,062**	**No n=1,875 (90.9%)**	**Yes n=187 (9.07%)**
Minority ethnic	986	910 (92.3%)	76 (7.7%)	1,558	1,404 (90.1%)	154 (9.9%)
Missing	458	441 (96.3%)	17 (3.7%)	20	20 (100.0%)	0 (0.0%)
**Geographical region[1]**						
East of England	4,889	4,611 (94.3%)	278 (5.7%)	319	297 (93.1%)	22 (6.9%)
London	2,044	1,830 (89.5%)	214 (10.5%)	889	769 (86.5%)	120 (13.5%)
South East	4,147	3,927 (94.7%)	220 (5.3%)	377	358 (95.0%)	19 (5.0%)
**IMD quintile**						
1 (most deprived)	1,679	1,526 (90.9%)	153 (9.1%)	230	211 (91.7%)	19 (8.3%)
2	2,948	2,730 (92.6%)	218 (7.4%)	436	375 (86.0%)	61 (14.0%)
3	4,297	4,054 (94.3%)	243 (5.7%)	473	435 (92.0%)	38 (8.0%)
4	5,675	5,334 (94.0%)	341 (6.0%)	449	417 (92.9%)	32 (7.1%)
5 (least deprived)	6,452	6,075 (94.2%)	377 (5.8%)	457	420 (91.9%)	37 (8.1%)

Boukari Y, *et al. J Epidemiol Community Health* 2023; 77:649–655. doi: 10.1136/jech-2022-220251

	UK-born			Migrant		
		Evidence of SARS-CoV-2 infection			Evidence of SARS-CoV-2 infection	
**Characteristic**	**Overall, N=21,416[1]**	**No n=20,074 (93.7%)**	**Yes n=1,342 (6.27%)**	**Overall, N = 2,062**	**No n=1,875 (90.9%)**	**Yes n=187 (9.07%)**
Missing	365	355 (97.3%)	10 (2.7%)	17	17 (100.0%)	0 (0.0%)
**Total household income**						
£0-9,999	712	666 (93.5%)	46 (6.5%)	94	81 (86.2%)	13 (13.8%)
£10,000-24,999	4,158	3,915 (94.2%)	243 (5.8%)	295	271 (91.9%)	24 (8.1%)
£25,000-49,999	6,570	6,140 (93.5%)	430 (6.5%)	510	474 (92.9%)	36 (7.1%)
£50,000-£74,999	3,173	2,914 (91.8%)	259 (8.2%)	395	360 (91.1%)	35 (8.9%)
£75,000-£99,999	1,504	1,391 (92.5%)	113 (7.5%)	222	198 (89.2%)	24 (10.8%)
£100,000+	1,410	1,308 (92.8%)	102 (7.2%)	315	277 (87.9%)	38 (12.1%)
Missing	3,889	3,740 (96.2%)	149 (3.8%)	231	214 (92.6%)	17 (7.4%)
**Household overcrowding status**						
Under-occupied	19,662	18,477 (94.0%)	1,185 (6.0%)	1,472	1,370 (93.1%)	102 (6.9%)
Balanced	1,320	1,206 (91.4%)	114 (8.6%)	366	315 (86.1%)	51 (13.9%)

Boukari Y, et al. J Epidemiol Community Health 2023; 77:649–655. doi: 10.1136/jech-2022-220251

	UK-born			Migrant		
		Evidence of SARS-CoV-2 infection			Evidence of SARS-CoV-2 infection	
**Characteristic**	**Overall, N=21,416[1]**	**No n=20,074 (93.7%)**	**Yes n=1,342 (6.27%)**	**Overall, N = 2,062**	**No n=1,875 (90.9%)**	**Yes n=187 (9.07%)**
Overcrowded	434	391 (90.1%)	43 (9.9%)	224	190 (84.8%)	34 (15.2%)
**Lives with any children**						
No	18,647	17,530 (94.0%)	1,117 (6.0%)	1,599	1,472 (92.1%)	127 (7.9%)
Yes	2,769	2,542 (91.8%)	227 (8.2%)	463	403 (87.0%)	60 (13.0%)
**Clinical vulnerability status**						
Not clinically vulnerable	11,715	10,946 (93.4%)	769 (6.6%)	1,249	1,123 (89.9%)	126 (10.1%)
Clinically vulnerable	5,846	5,457 (93.3%)	389 (6.7%)	549	513 (93.4%)	36 (6.6%)
Clinically extremely vulnerable	2,470	2,345 (94.9%)	125 (5.1%)	244	219 (89.8%)	25 (10.2%)
Missing	1,385	1,326 (95.7%)	59 (4.3%)	20	20 (100.0%)	0 (0.0%)

Supplementary Figure 1: Directed acyclic graph of the relationship between migration status and SARS-CoV-2 infection

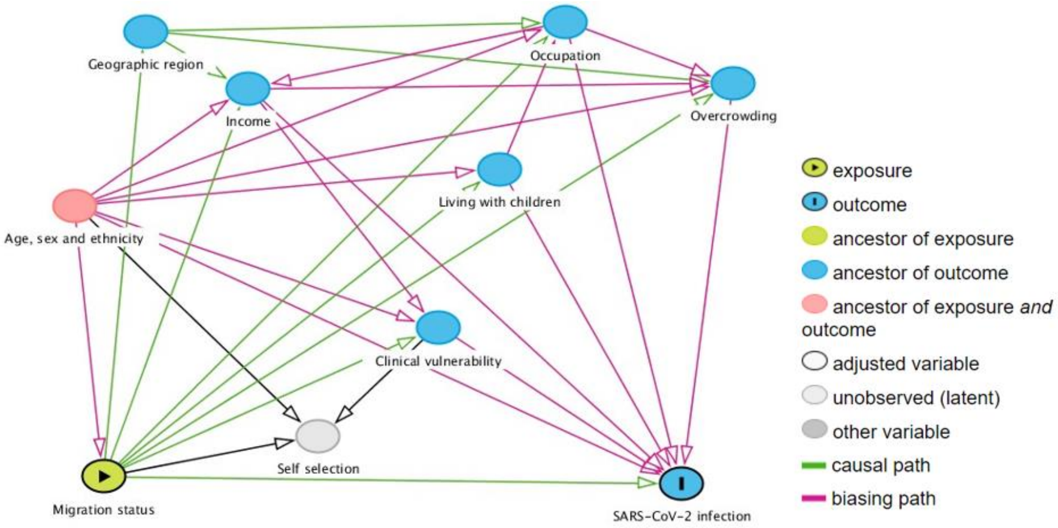

Boukari Y, *et al. J Epidemiol Community Health* 2023; 77:649–655. doi: 10.1136/jech-2022-220251

Supplementary Figure 2: Counts for different person per room (PPR) values across the study cohort

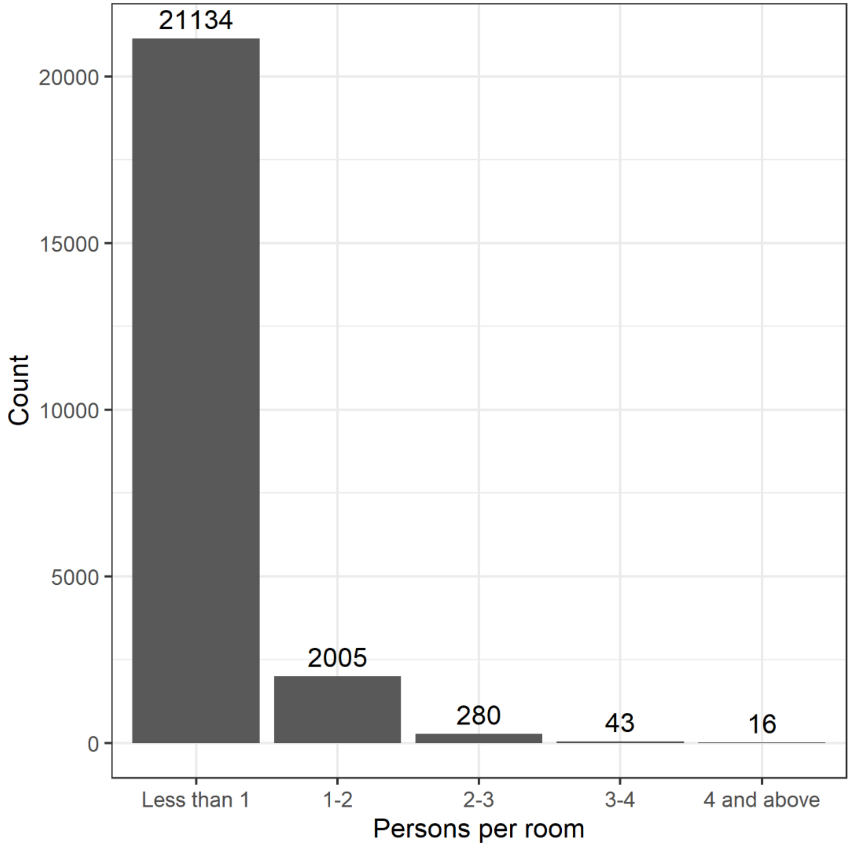

References

1 Hernán MA, Robins JM. Causal Inference: What If: Chapman & Hall/CRC; 2020.
2 Buis ML. Direct and indirect effects in a logit model. Stata Journal. 2010;**10**(1):11-29.
3 Beale S, Patel P, Rodger A, Braithwaite I, Byrne T, Fong WLE, et al. Occupation, work-related contact and SARS-CoV-2 anti-nucleocapsid serological status: findings from the Virus Watch prospective cohort study. Occupational and Environmental Medicine. 2022;**Epub ahead of print**.
4 Fox J, Weisberg S. An R Companion to Applied Regression. Third edition ed. Thousand Oaks CA: Sage; 2019.
5 Fox J, Monette G. Generalized Collinearity Diagnostics. J Am Stat Assoc. 1992;**87**(417):178-83.
6 O'Brien RM. A caution regarding rules of thumb for variance inflation factors. Qual Quant. 2007;**41**(5):673-90.

Boukari Y, *et al. J Epidemiol Community Health* 2023; 77:649–655. doi: 10.1136/jech-2022-220251
